# Assessing the reporting quality of published qualitative evidence syntheses in the cochrane library

**DOI:** 10.1002/cesm.70023

**Published:** 2025-04-15

**Authors:** Martina Giltenane, Aoife O'Mahony, Mayara S. Bianchim, Andrew Booth, Angela Harden, Catherine Houghton, Emma F. France, Heather Ames, Kate Flemming, Katy Sutcliffe, Ruth Garside, Tomas Pantoja, Jane Noyes

**Affiliations:** ^1^ School of Nursing and Midwifery, Health Research Institute University of Limerick Limerick Ireland; ^2^ School of Public Health University College Cork Cork Ireland; ^3^ School of Health Sciences Bangor University Bangor UK; ^4^ School of Health and Related Research University of Sheffield Sheffield UK; ^5^ School of Health and Medical Sciences, City St George's University of London London UK; ^6^ School of Nursing and Midwifery University of Galway Galway Ireland; ^7^ Centre for Healthcare and Community Research, Faculty of Health Sciences and Sport University of Stirling Stirling UK; ^8^ Division for Health Services Global Health Department Oslo Norway; ^9^ Department of Health Sciences The University of York York UK; ^10^ EPPI Centre, Social Research Institute University College London London; ^11^ European Centre for Environment and Human Health University of Exeter Penryn UK; ^12^ Department of Family Medicine, School of Medicine Pontificia Universidad Catolica de Chile Santiago USA

**Keywords:** mixed‐methods reviews, qualitative evidence synthesis, quality assessment, reporting quality

## Abstract

**Background:**

Over ten years since the first qualitative evidence synthesis (QES) was published in the Cochrane Library, QES and mixed‐methods reviews (MMR) with a qualitative component have become increasingly common and influential in healthcare research and policy development. The quality of such reviews and the completeness with which they are reported is therefore of paramount importance.

**Aim:**

This review aimed to assess the reporting quality of published QESs and MMRs with a qualitative component in the Cochrane Library.

**Methods:**

All published QESs and MMRs were identified from the Cochrane Library. A bespoke framework developed by key international experts based on the Effective Practice and Organisation of Care (EPOC), Enhancing Transparency in Reporting the Synthesis of Qualitative Research (ENTREQ) and meta‐ethnography reporting guidance (eMERGe) was used to code the quality of reporting of QESs and MMRs.

**Results:**

Thirty‐one reviews were identified, including 11 MMRs. The reporting quality of the QESs and MMRs published by Cochrane varied considerably. Based on the criteria within our framework, just over a quarter (8, 26%) were considered to meet satisfactory reporting standards, 10 (32%) could have provided clearer or more detailed descriptions in their reporting, just over a quarter (8, 26%) provided poor quality or insufficient descriptions and five (16%) omitted descriptions relevant to our framework.

**Conclusion:**

This assessment offers important insights into the reporting practices prevalent in these review types. Methodology and reporting have changed considerably over time. Earlier QES have not necessarily omitted important reporting components, but rather our understanding of what should be completed and reported has grown considerably. The variability in reporting quality within QESs and MMRs underscores the need to develop Preferred Reporting Items for Systematic Reviews and Meta‐Analyses (PRISMA) specifically for QES.

## Highlights

QESs and mixed‐methods reviews (those including data from both quantitative and qualitative research).

## 1 INTRODUCTION

Qualitative evidence synthesis (QES) enables researchers to synthesise evidence from primary qualitative studies to develop new cumulative knowledge [1]. This process is pivotal for comprehending experiences, perspectives, beliefs, and priorities, as well as for developing clinical guidelines [1]. QES can provide invaluable insights for decision‐makers regarding context, stakeholder experiences, and intervention implementation. However, such approaches are not without challenges and generate considerable methodological uncertainty [2]. In recent years, the Cochrane Qualitative and Implementation Methods Group (QIMG) has been instrumental in raising awareness of, and providing guidance for, the use of QES methods [3, 4]. Nevertheless, the value of QES depends not only on the rigour of the synthesis process but also on the clarity and comprehensiveness of its reporting. High‐quality reporting ensures transparency, reproducibility, and credibility, which are essential for evidence synthesis to inform practice and policy effectively [5].

Reporting guidelines ensure authors provide sufficient information to enable an understanding of how the review was conducted, thereby helping to facilitate an assessment of quality of QES conduct [6]. PRISMA (Preferred Reporting Items for Systematic Reviews and Meta‐Analyses; [7]) outlines reporting guidelines for quantitative systematic reviews, however, researchers conducting QESs face a gap in the absence of applicable and up‐to‐date guidelines. Although PRISMA is widely used and is considered high quality, it is not entirely suitable for QES. While the ENTREQ (Enhancing Transparency in Reporting the Synthesis of Qualitative Research) [8] guideline is widely used, significant advancements in QES methods have occurred since its publication, more than a decade ago. Similarly, while meta‐ethnography reporting guidance (eMERGe) [9] provides evidence‐based reporting guidance, it is not designed for other forms of QES.

In recent times, reviewers have used the EPOC (Effective Practice and Organisation of Care) template [10] to guide the reporting of their QES. While this template incorporates valuable guidance, its adoption has not been consistent and has only been available relatively recently as a web‐based resource with publication in a journal article in 2021. Understanding the characteristics of QESs published by Cochrane could inform the creation of a QES specific reporting guideline. It is timely to examine the reporting quality of QESs and MMRs with a qualitative component as they have become increasingly used to guide policy and clinical guideline development in healthcare research [11]. High‐quality reporting is critical to ensuring that evidence syntheses are useful and reliable for decision‐making and knowledge translation. With the first QES published in the Cochrane Library in 2013 [12], now, more than a decade later, it is timely to examine the reporting quality of such reviews. By addressing the gaps in reporting standards, this review aims to inform future improvements in reporting guidance for QESs and MMRs.

## 2 METHODS

A review and assessment of reporting quality of QESs and MMRs published by the Cochrane Library was conducted using a bespoke composite framework (Figure 1) drawing on key components of EPOC, ENTREQ and eMERGe frameworks and international expert input and agreement. To ascertain agreement, similar methods used by Ames et al. (2024) were employed. MG and AOM designed the composite framework drawing on key reporting components of EPOC, ENTREQ and eMERGe reporting guidance. The new composite framework was then presented to the wider QIMG convenors for their input and agreement on all indicators within each domain. Of note the aforementioned convenors were highly experienced QES reviewers and methodologists with an interest in reporting quality, several of whom had developed the original reporting guidelines and authored chapters in the new Cochrane‐Campbell Handbook for QES.

Figure 1
**Framework guided by the former EPOC Guidelines, ENTREQ and eMERGe frameworks for reporting and methodological quality and international input and agreement**.

**Figure:**
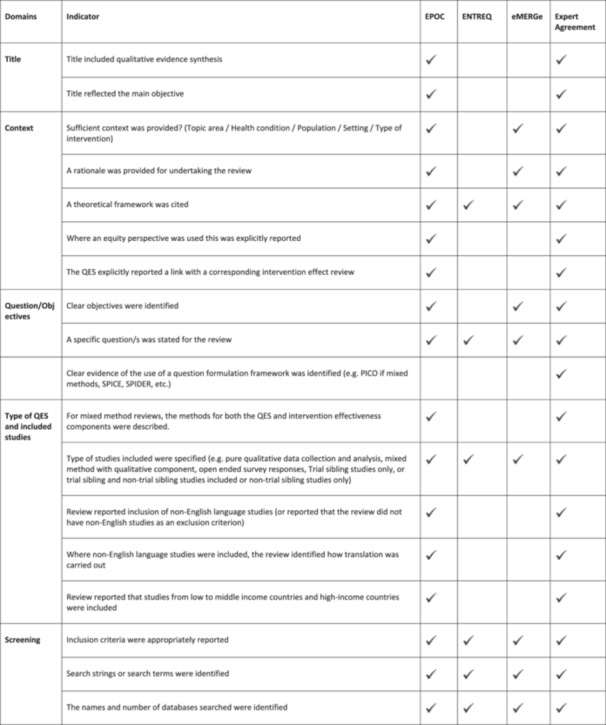

**Figure:**
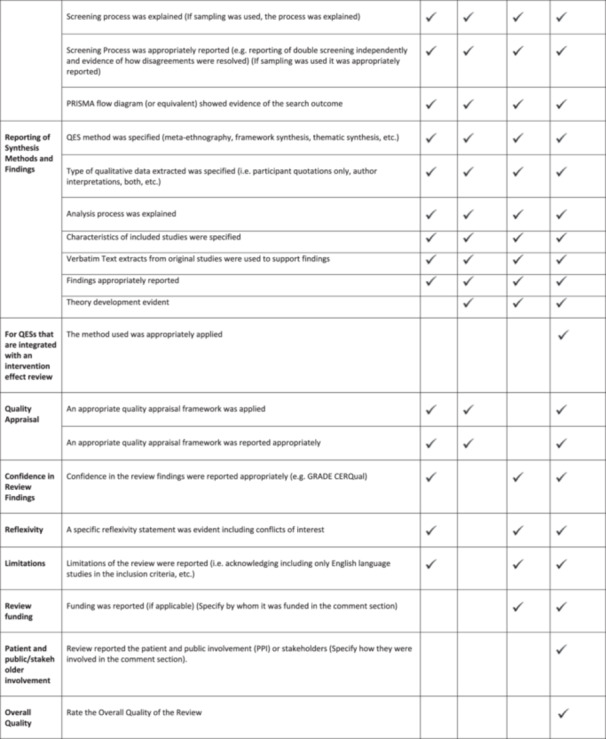

### 2.1 Review question

This review aimed to elucidate the following question: How well are qualitative evidence syntheses and mixed‐methods reviews with a qualitative component reported in the Cochrane Library?

### 2.2 Search strategy

All published QESs and MMRs up to August 2023 were identified from the Cochrane Library using the search string shown in Table 1.

**Table 1:** Search String.

*Qualitative OR “mixed method” OR meta‐synthesis OR “qualitative evidence synthesis” OR “framework synthesis” OR meta‐ethnography OR “thematic synthesis” OR realist OR “qualitative comparative analysis”*

### 2.3 Inclusion criteria

We included any review that synthesised findings from qualitative primary studies included in the Cochrane library from 2013 to 2023 including QESs and MMRs with a qualitative component.

### 2.4 Screening process

Two reviewers (MG and AOM) independently reviewed titles and abstracts using PICO Portal, a system for housing and screening studies [13]. Studies meeting inclusion criteria were included for full‐text screening. MG and AOM independently screened the full texts. Any discrepancies in screening judgements were resolved by discussion between the two reviewers through re‐reviewing the review in collaboration and agreeing a judgement, a third reviewer was not required during the screening process.

### 2.5 Data collection and analysis

**Figure 2:**
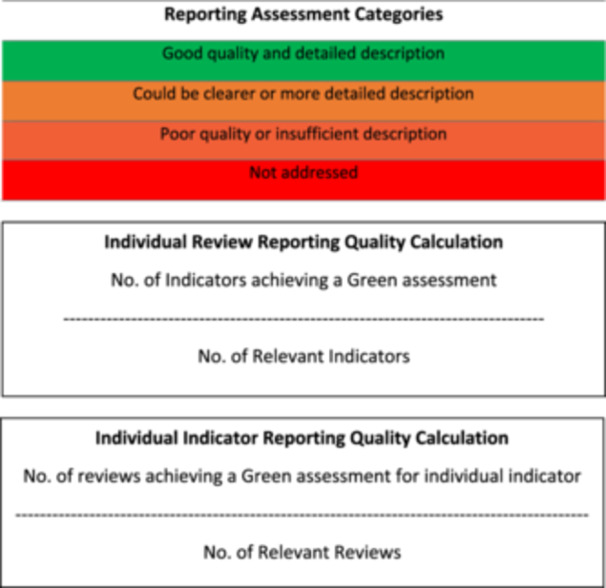
**Reporting Assessment Categories and Calculation**.

Using the bespoke composite framework the reporting quality of reviews was assessed. Three reviewers (MG, AOM and MSB) independently assessed and coded one review at the beginning of the process to ensure consistency. Disagreements in the coding were resolved through discussion between the three reviewers. All data extracted from each review were assessed by two of the three reviewers (MG, AOM, MSB). Data analysis was conducted by three team members (MG, AOM, MSB) in the form of descriptive statistics. After careful consideration and discussion amongst co‐authors, keeping in line with qualitative processes generally, the aim was to achieve consensus and calibration amongst reviewers and not to specifically ‘validate’ the specific criteria. Therefore, reporting quality of the reviews are presented using a traffic light colour coded system. This process aligns with other qualitative systematic review processes such as assessment of methodological limitations in primary studies, whereby reviewers make their own assessments and then agree by consensus. Calculating inter‐rater reliability is not considered appropriate in this context. Thus, instead of rigid percentage cut off parameters, we have presented our findings based on consensus of the qualitative criteria. The levels of achievement were qualitatively assessed and considered as 1) having a good quality and detailed description (green), 2) could have provided clearer or more detailed descriptions (amber), 3) considered poor quality or insufficient descriptions (amber) or 4) omitted descriptions required by our framework criteria altogether (red). Through discussion and agreement with the wider QIMG, the research team considered these parameters and the traffic light colour coded system a practical way to simplify the readability of results. An example of the criteria used for assessing one Indicator is presented in Table 2. The reporting quality of individual reviews was calculated based on the number of indicators achieving a green assessment ‘good quality and detailed description’ by the review team divided by the number of indicators relevant to each review and converted to percentages. Where it was not relevant to include the reporting of individual indicators in particular review types, these were judged as N/A and not included in the calculation (Figure 2). For example, QESs were not included in the percentage calculation for specific items related to mixed‐methods reviews. Similarly, the quality of reporting of individual indicators across all reviews was calculated based on the number of reviews achieving a green assessment ‘good quality and detailed description’ by the review team for each indicator divided by the number of relevant reviews and converted to percentages (Figure 2).

**Table 2:** Example of Assessment Categories Used for ‘How Findings Section was Reported’.

Criteria	RAG Status
The product of the synthesis method is not reported ‐ for example over‐reliance on only reporting summarised statements to which GRADE‐CERQual can be applied. These truncated findings may be organised under headings.	
The product of the synthesis method is reported: Themes but no quotations to support findings	
The product of the synthesis method is reported: Themes and some quotations but without much interpretation, followed by summarised findings statements to which GRADE‐CERQual can be applied.	
The product of the synthesis method is reported: Themes, quotations, interpretation, followed by summarised findings statements to which GRADE‐CERQual can be applied. New theoretical insights or theory might have been produced.	

## 3 FINDINGS

**Figure 3:**
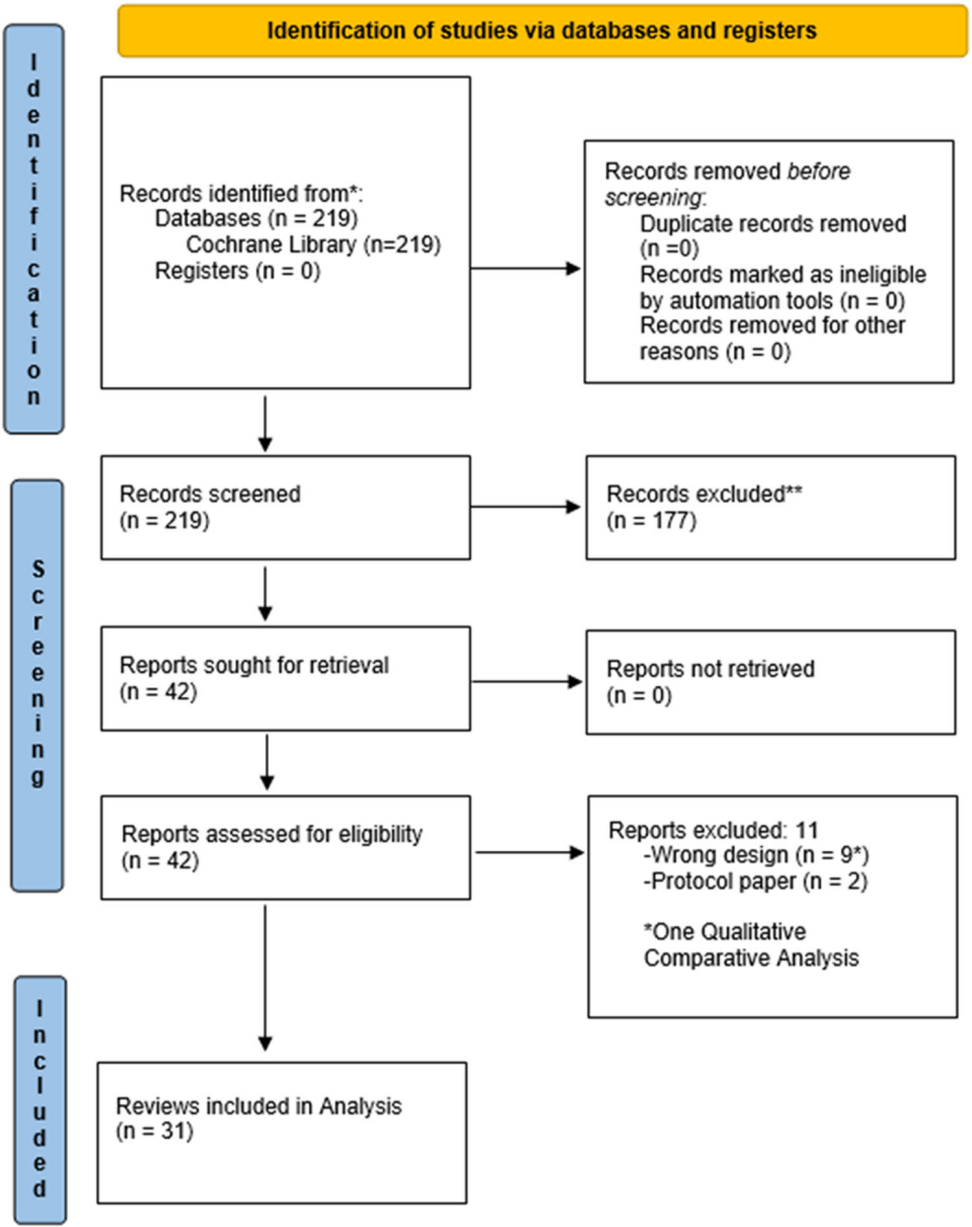
**PRISMA flow‐chart**.

**Figure 4:**
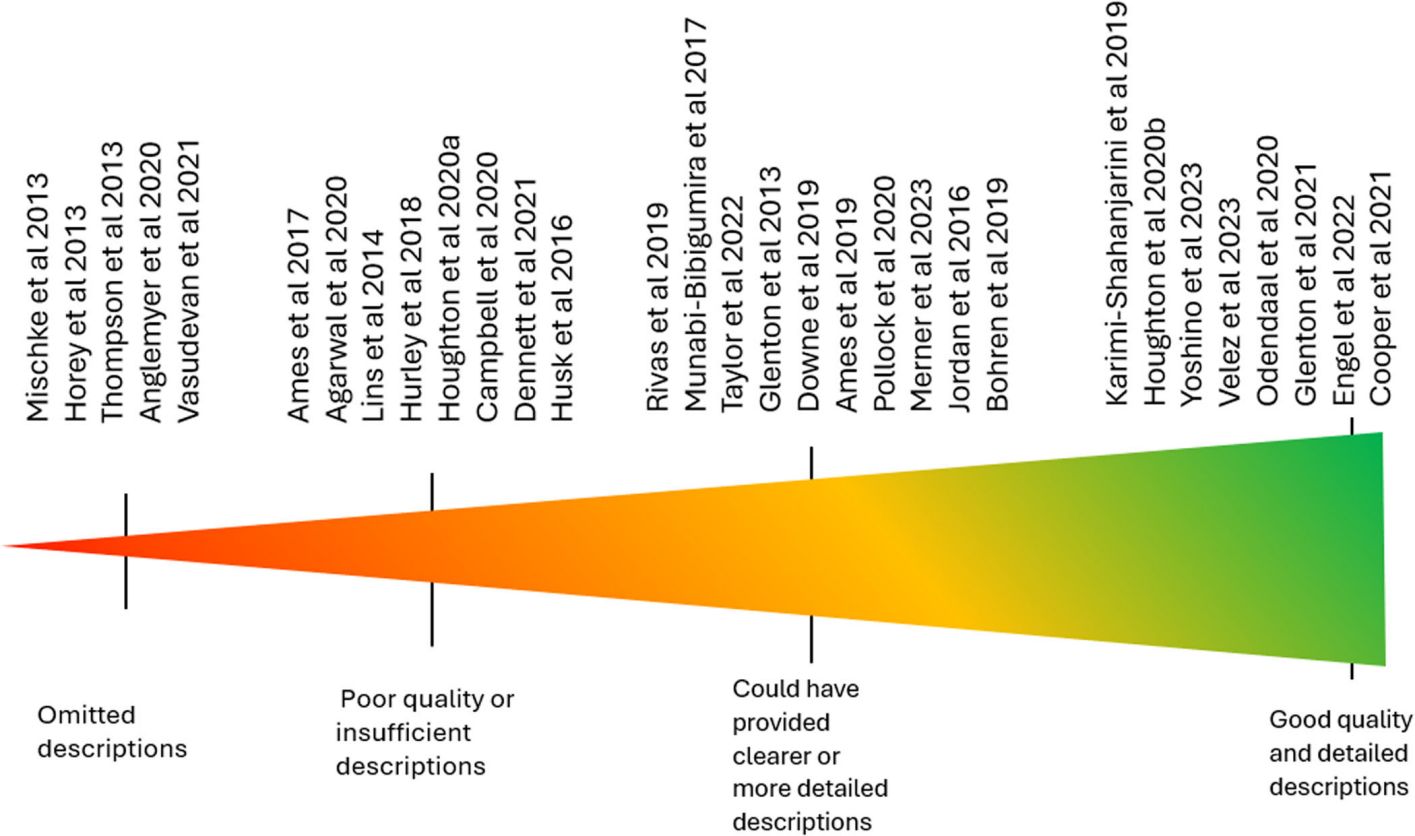
**Reporting Quality of Individual Reviews against Reporting Framework.** (Figure adapted from [44]).

**Figure 5:**
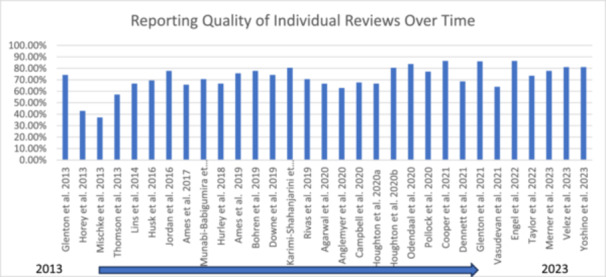
**Reporting Quality of Individual Reviews Over Time**.

In total, 219 titles were initially identified from the Cochrane Library. All 219 titles and abstracts were screened, and 42 reviews proceeded to full‐text screening. Ten reviews were excluded during full‐text screening and reasons recorded, primarily due to the reviews not synthesising qualitative evidence (Figure 3). A Qualitative Comparative Analysis (QCA) was assessed as inappropriate due to its mathematical function to systematically compare cases and derive solutions [14]. This study was removed from data extraction and analysis. As a result, 31 reviews were included comprising 20 QESs and 11 MMRs. Table 3 outlines the characteristics of the included reviews. The reporting quality of the reviews varied considerably and when assessed against the criteria within our framework, over a quarter (n = 8; 26%) of the reviews were considered as having good quality and detailed descriptions (green), 10 (32%) could have provided clearer or more detailed descriptions (amber), eight (26%) provided poor quality or insufficient descriptions (amber) and five (16%) omitted descriptions (red) (Figure 4). Figure 5 shows the reporting quality of individual reviews over time.

**Table 3:** Characteristics of Included Studies.

Author & year	Type of review (mixed‐methods, QES)	Settings and population	QES method	Types of studies included in the review	Sample (No. of studies included)	Study focus
[15]	QES	Clients in the areas of reproductive, maternal, newborn, child, or adolescent health from any setting globally	Framework thematic synthesis	Studies that used qualitative methods for data collection and analysis	35	To explore clients' perceptions and experiences of targeted digital communication via mobile devices on topics related to reproductive, maternal, newborn, child, or adolescent health (RMNCAH).
[16]	Mixed method	Primary Healthcare Healthcare workers	Thematic synthesis	Mainly case studies, randomised trials and mixed method (before and after and interviews)	16	To assess the effects of strategies for notifying stock levels and digital tracking of healthcare‐related commodities and inventory via mobile devices across the primary healthcare system.
[17]	QES	Parents and informal caregivers from any setting globally where information about childhood vaccinations was communicated or distributed	Thematic synthesis	Studies that used qualitative methods for data collection and analysis	38	Views and experiences of parents and informal caregivers regarding information about vaccination for children aged up to 6 years.
[18]	Mixed method	People involved in digital contact tracing during infectious disease outbreaks in any setting.	Thematic synthesis	Cohort studies and modelling studies	12	To assess the benefits and harms of digital solutions for identifying contacts of an identified positive case of an infectious disease and to assess acceptability of this approach from qualitative studies
[19]	QES	Women, partners, family members, doulas, providers, or other relevant stakeholders' Any type of health facility in any setting globally	Thematic synthesis	Studies that used qualitative methods for data collection and analysis	52	To describe and explore the perceptions and experiences of women, partners, community members, healthcare providers and administrators, and other key stakeholders regarding labour companionship; to identify factors affecting successful implementation and sustainability of labour companionship; and to explore how the findings of this review can enhance understanding of the related Cochrane systematic review of interventions.
[20]	QES	Women who had smoked in pregnancy.	Thematic synthesis	Studies that used qualitative methods for data collection and analysis	21	To explore factors affecting uptake and use of nicotine replacement therapy and e‐cigarettes in pregnancy
[21]	QES	Parents and informal caregivers Diverse geographical settings, and from a range of income‐level settings.	Meta ethnography	Studies that used qualitative methods for data collection and analysis	53	Explore parents' and informal caregivers' views and practices regarding routine childhood vaccination, and the factors influencing acceptance, hesitancy, or nonacceptance of routine childhood vaccination.
[22]	Mixed method	Patients, carers and healthcare professionals receiving or providing care to manage COPD	One qualitative study only, unable to apply QES methods	Mainly randomised trials, mixed method and qualitative	7	To assess the effectiveness of any single intervention for COPD adapted or tailored to their comorbidity(s) compared to any other intervention for people with COPD and one or more common comorbidities (quantitative data, randomised trials) To describe the views and experiences of patients, carers and healthcare professionals when receiving or providing care to manage multi‐morbidities.
[23]	QES	Pregnant or postnatal women. Healthcare providers, including lay or community health workers, in rural and urban locations globally.	Meta‐ethnographic and framework techniques	Studies that used qualitative methodology	85	To explore women's and healthcare workers' views and experiences of antenatal care.
[24]	QES	People with presumptive or confirmed tuberculosis and drug‐resistant tuberculosis and their caregivers, healthcare providers, laboratory technicians and managers, and programme officers and staff from any type of health facility and setting globally.	Thematic synthesis	Studies that used qualitative methods for data collection and analysis	32	To synthesize end‐user and professional user perspectives and experiences with low‐complexity nucleic acid amplification tests (NAATs) for detection of tuberculosis and tuberculosis drug resistance; and to identify implications for effective implementation and health equity.
[12]	QES	Health professionals in primary or community healthcare setting	Framework thematic synthesis approach	Studies that used qualitative methods for data collection and analysis a	53	To explore factors affecting the implementation of Lay Health Workers programmes for maternal and child health.
[25]	QES	Healthcare workers including doctors, nurses, pharmacists and others working in hospitals, clinics, pharmacies and nursing homes	Thematic synthesis approach	Qualitative studies and mixed‐methods studies with an identifiable qualitative component	11	To explore healthcare workers' perceptions and experiences of communicating with older adults about vaccination.
[26]	Mixed method	pregnant women who have previously had a caesarean birth and health professionals	Narrative synthesis	randomised controlled trials (RCTs) and quasi‐randomised trials and one qualitative study	4	To examine the eIectiveness of interventions to support decision‐making about vaginal birth after a caesarean birth
[27]	QES	Healthcare workers with responsibility for patient care in hospitals and primary and community care settings	‘best fit framework approach’ to analyse and synthesise the evidence.	qualitative and mixed‐methods studies (with an identifiable qualitative component)	20	To identify barriers and facilitators to healthcare workers' adherence to IPC guidelines for respiratory infectious diseases
[28]	QES	Potential trial participants over 18 who were not assessed as having impaired mental capacity	Thematic synthesis	Qualitative and mixed‐methods studies (with an identifiable qualitative component)	29 studies (30 papers)	To explore potential trial participants' views and experiences of the recruitment process for participation.
[29]	Mixed method	Participants aged 45 years or older, with a clinical diagnosis of osteoarthritis or self‐reported chronic hip or knee (or both) pain (defined as more than six months' duration).	Framework synthesis	Randomised control trials and qualitative studies	33 (21 quantitative and 12 qualitative studies)	To improve our understanding of the complex inter‐relationship between pain, psychosocial effects, physical function and exercise
[30]	Mixed method	Adults in urban or rural locations in any country	Thematic synthesis	Randomised controlled trials (RCTs), cluster RCTs, quasi‐RCTs, cluster quasi‐RCTs, controlled before‐and‐after studies, interrupted‐time‐series, cohort studies (prospective or retrospective), case‐control studies and uncontrolled before‐and‐after studies (uBA). Qualitative research that used recognised qualitative methods of data collection and analysis.	19 studies (28 publications of which 9 qualitative, 7 quantitative and 3 mixed methods)	To assess the health and well‐being impacts on adults following participation in environmental enhancement and conservation activities.
[31]	QES	Healthcare professionals either involved in the design, implementation or use of weaning protocols or involved in the weaning of critically‐ill adults and children from mechanical ventilation not using protocols	Thematic synthesis	Qualitative studies	11	To locate, appraise and synthesize qualitative evidence concerning the barriers and facilitators of the use of protocols for weaning critically‐ill adults and children from mechanical ventilation
[32]	QES	Doctors, nurses, patients and their families/carers, policymakers, programme managers, other health workers and any others directly involved in or affected by doctor‐nurse substitution	Framework thematic synthesis approach	Studies that had collected and analysed qualitative data	66 studies (69 papers)	To identify factors influencing implementation of interventions to substitute doctors with nurses in primary care.
[33]	Mixed method	Informal carers of people with dementia	Thematic synthesis	Randomised controlled trials (RCTs) or cross‐over trials and qualitative studies with qualitative methods of data collection and analysis.	11 (9 RCTs and 2 Qualitative studies)	The efficacy of telephone counselling for informal carers of people with dementia; Synthesize qualitative studies to explore carers' experiences of receiving telephone counselling and counsellors' experiences of conducting telephone counselling
[34]	QES	Patients, carers or community member and people with a health policy, management, administrative or clinical role who participate in formal partnerships in an advisory or representative capacity	Framework synthesis	Qualitative studies	33	To synthesise the views and experiences of consumers and health providers of formal partnership approaches that aimed to improve planning, delivery or evaluation of health services and to identify best practice principles for formal partnership approaches in health services by understanding consumers' and health providers' views and experiences.
[35]	Mixed method	Studies of firms or workplaces evaluating inspections, warnings or orders, citations or fines, prosecution or firm closure by governmental representatives and if the outcomes were injuries, diseases or exposures.	Not mentioned	Randomised controlled trials (RCTs), controlled before‐after studies (CBAs), interrupted time series (ITS), econometric panel studies and qualitative studies.	23 (6 qualitative studies)	To assess the effects of occupational safety and health regulation enforcement tools for preventing occupational diseases and injuries.
[47]	QES	Studies from all levels of health care and included doctors, midwives, nurses, auxiliary nurses and their managers.	Best fit framework synthesis approach	Qualitative studies that focused on views, experiences, and behaviours.	31	To explore the views, experiences, and behaviours of skilled birth attendants and those who support them; to identify factors that influence the delivery of intrapartum and postnatal care in low‐ and middle‐income countries; and to explore the extent to which these factors were reflected in intervention studies
[36]	QES	Studies from public or private primary healthcare facilities, community and workplace, or the homes of clients. All categories of health workers, as well as those persons who supported the delivery and management of the mHealth programmes (excluding technical staff).	Thematic synthesis	Studies that used qualitative data collection and analysis methods.	43	To synthesise qualitative research evidence on health workers' perceptions and experiences of using mHealth technologies to deliver primary healthcare services, and to develop hypotheses about why some technologies are more effective than others.
[37]	Mixed method	Studies in which participants were health and social care professionals working at the front line during infectious disease outbreaks, categorised as epidemics or pandemics by WHO, from 2002 onwards.	Best fit framework synthesis	Randomised trials, non‐randomised trials, controlled before‐after studies, interrupted time series studies, and qualitative and descriptive studies	16	To assess the effects of interventions aimed at supporting the resilience and mental health of frontline health and social care professionals during and after a disease outbreak, epidemic or pandemic and to identify barriers and facilitators that may impact on the implementation of interventions aimed at supporting the resilience and mental health of frontline health and social care professionals during and after a disease outbreak, epidemic or pandemic.
[38]	QES	Empirical studies of any advocacy or multi‐component intervention including advocacy, intended for women aged 15 years and over who were experiencing or had experienced any form of intimate partner abuse, or of advocates delivering such interventions, or experiences of women who were receiving or had received such an intervention.	Realist approach	Survey‐based, instrument development, qualitative studies, experimental intervention studies (some including qualitative evaluations). randomised controlled trials, mixed methods studies.	98 studies (148 articles)	To assess advocacy interventions for intimate partner abuse in women, in terms of which interventions work for whom, why and in what circumstances.
[39]	QES	Studies explored community members and community drug distributors' experiences, perceptions, or attitudes towards Mass Drug Administration programmes for lymphatic filariasis in any country	Thematic synthesis	Qualitative research and mixed‐methods studies when it was possible to extract qualitative data	29	To synthesize qualitative research evidence about community experience with, and understanding and perception of, Mass Drug Administration programmes for lymphatic filariasis and to explore whether programme design and delivery influence the community experience identified in the analysis.
[40]	Mixed method	Studies which assessed change in any health outcome of residents following housing improvement were included. All housing improvements which involved a physical improvement to the fabric of the house were included.	Narrative synthesis	Randomised controlled trials, nonexperimental studies, 12 nonexperimental studies. Studies reporting qualitative data. The included qualitative studies also reported quantitative data which was included in the review	39	To assess the health and social impacts on residents following improvements to the physical fabric of housing.
[41]	Mixed method	Studies focussed on identification of births and deaths in rural, remote, or marginalised populations who are typically underrepresented in civil registration processes or traditionally seen as having poor access to health services and included lay health workers, family members, healthcare organisations, and community‐based informants.	Framework synthesis	Before‐after study, and quantitative, qualitative, and descriptive designs.	21	To assess the effects of birth notification and death notification via a mobile device, compared to standard practice. And to describe the range of strategies used to implement birth and death notification via mobile devices and identify factors influencing the implementation of birth and death notification via mobile devices.
[42]	QES	Studies that explored patients, caregivers, healthcare providers and family members about the provision of in‐person home‐based rehabilitation and home‐based telerehabilitation services.	Framework thematic synthesis using the CFIR (Consolidated Framework for Implementation Research) framework	Studies that used qualitative methods for data collection and analysis	53	To identify factors that influence the organisation and delivery of in‐person home‐based rehabilitation and home‐based telerehabilitation for people needing rehabilitation.
[43]	QES	Recipients of cash transfer interventions where health outcomes were evaluated including adult patients of healthcare services, the general adult population as recipients of cash targeted at themselves or directed at children from any country.	Meta ethnography	Qualitative methods or mixed‐methods studies with qualitative research	41	To explore how conditional and unconditional cash transfer social protection interventions with a health outcome are experienced and perceived by their recipients

Three of the four reviews published in 2013 provided poor‐quality descriptions, insufficient descriptions or omitted descriptions when assessed against the criteria in our framework [26, 35], Thompson et al., 2013). This may reflect the lack of available reporting guidelines at that time. However, despite limited guidance one review published in 2013 [12] made a good attempt to provide descriptions of criteria within our framework, albeit they could have been somewhat clearer in their reporting. Furthermore, two recent reviews, published when all the guidance tools underpinning our framework were available, were considered to have omitted descriptions of criteria within our framework altogether. For example, these reviews did not include verbatim text extracts in their reporting of their findings, lacked theory development or inclusion of patient and public involvement (PPI) [18, 41].

### 3.1 Reporting context and the use of an appropriate and informative title

Twenty‐six (84%) reviews specified in the title whether the review was a QES or MMR, and one (3%; [18]) identified itself as a rapid review but did not specify it had a qualitative component. While the remaining four (13%; [26, 33, 35, 40]) did not specify any type of review in their titles; these four reviews were MMRs published between 2013 and 2014, when standards for reporting were less established. In total, 27 (87%) review titles reflected their main objective, but this was less clear in four reviews (13%) [16, 18, 29, 39].

Twenty‐three reviews (74%) provided sufficient background context for the review topic; however, the remaining eight reviews (26%) required clearer or greater detail. Twenty‐eight reviews (90%) provided a rationale for the review, but this could have been clearer or more detailed in the remaining three reviews (10%; [18, 24, 29]). For example, while Hurley et al. [29] included a section titled ‘Why is it important to do this review,’ this section identified the review questions as opposed to reporting the rationale for conducting the review.

**Figure 6:**
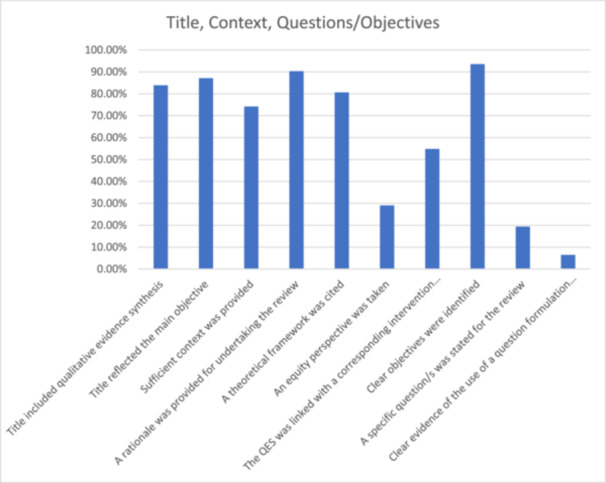
**Percentage of reviews that achieved a green assessment for indicators relating to title, context, questions/objectives**.

Twenty‐five reviews presented a clear theoretical framework (81%), but this aspect could have been clearer in six reviews (19%). An equity perspective (i.e., considering the topic, methodology and/or results through the lens of potential unequal impacts across different populations or contexts) was adopted in nine reviews (29%), this could have been clearer or more detailed in 12 reviews (39%) and was not present in the remaining 10 reviews (32%). Seventeen (55%) QES were clearly linked to a corresponding intervention effect review, but this could have been clearer or more detailed in four reviews (13%) and was absent in the remaining 10 (32%) (Figure 6).

### 3.2 Reporting research question and objectives

Clear objectives were identified in almost all reviews (*n* = 29, 94%) and only two needed additional detail (6%; [29, 39]). However, only six reviews included a specific review question (19%; [18, 21, 24, 29, 31, 38]) with only two (6%) reported using a question formulation framework [18, 31] (Figure 6).

### 3.3 Reporting types of included studies

Twenty‐eight reviews (90.3%) specified the type of studies included (e.g. purely qualitative data collection and analysis, mixed method with qualitative component, open‐ended survey responses, trial sibling studies only, or trial sibling and non‐trial sibling studies included or non‐trial sibling studies only). For example, Ames et al. [17] clearly described how they included studies that utilised qualitative methods for both data collection and analysis. Two (6.5%) reviews could have provided a clearer or more detailed description of the studies they included [29, 30]. For instance, Hurley et al. [29] did not summarise the types of study designs of included qualitative studies in the main text, only mentioning these in tables in the appendices. One review (3.2%) did not report the type of studies included in their review [38].

Regarding language of included primary studies, eighteen reviews (58%) reported attempting to include non‐English language studies or did not have non‐English language studies as an exclusion criterion. Agarawal et al. (2020) clearly reported including studies regardless of publication language. One review (3%) required more detail regarding inclusion of non‐English studies (Cooper et al., 2020). Twelve reviews (39%) did not report attempting to include non‐English language studies. Thompson (2013) acknowledged that inclusion of non‐English language studies was beyond their resources.

Among the 18 reviews attempting to include non‐English language studies, 16 included non‐English language studies with 13 (81%) identifying how translation was carried out. Two (13%) did not specify how translation was carried out [16, 22] and one (6%) could have provided more clarity [31].

**Figure 7:**
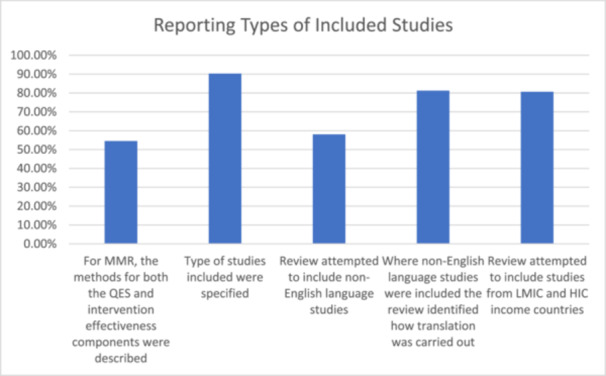
**Percentage of reviews that achieved a green assessment for indicators relating to the reporting of type of included studies**.

Twenty‐five (81%) reviews reported that they attempted to include studies from low‐to middle‐income countries (LMIC) and high‐income countries (HIC). One review (3%) could have provided more detail as to how they attempted to include studies from LMIC or HIC [37]. Five reviews (16%) did not clearly report the intention to include LMIC and/or HIC [26, 33, 35, 38], Thompson et al., 2013) (Figure 7).

### 3.4 Reporting search strategy and screening

Inclusion criteria were specified in 29 reviews (94%), two (6%) could have provided more detail when describing their inclusion criteria [17, 27]. For example, Ames et al. [17] provided a lengthy list of the vaccines and combinations of vaccines they included in the search; however specific inclusion criteria could have been made more explicit.

All reviews (*n* = 31; 100%) provided a complete description of the databases searched. However, only 28 (90.3%) were considered to have clearly reported their search strategy. Two (6.5%) provided insufficient detail of their search strategy [17, 26], such that the terms and/or combinations used were not considered to be reproducible. One review (3.2%) could have been clearer in their description of their search strategy used (e.g. including strings and terms) [35].

All reviews (*n* = 31; 100%) included a flow diagram visually summarising the screening process. Most reviews (*n* = 30; 97%) provided a good explanation of the screening process used, with one exception [35]. While Mischke et al. [35] identify duplicate ‘checking’ of full texts against inclusion criteria, their description mainly focussed on the screening of reference lists and inclusion of additional studies as opposed to the screening process of titles, abstracts and full text completed by the team.

**Figure 8:**
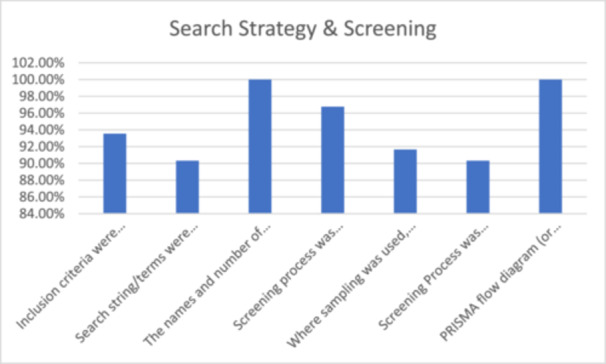
**Percentage of reviews that achieved a green assessment for individual indicators relating to search strategy and screening**.

Of 12 reviews (39%) that used a purposive sampling approach [1], 11 (92%) provided a clear explanation of their sampling process. Rivas et al., [38] mentioned sampling until theoretical saturation was reached but the description of this process was generic (Figure 8).

### 3.5 Reporting synthesis methods

**Figure 9:**
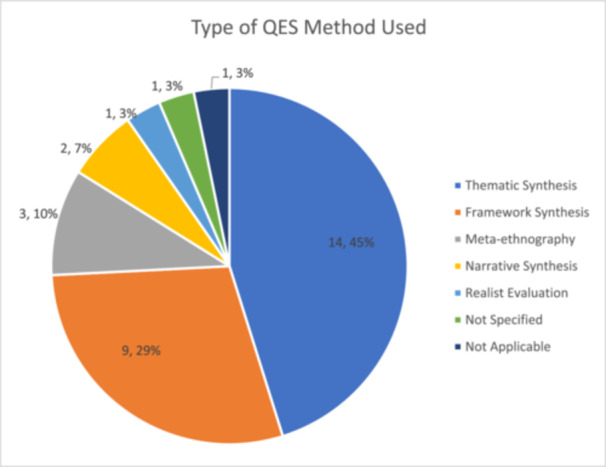
**Types of QES method used**.

The type of synthesis method used was clearly reported in 27 (90%) reviews. Thematic synthesis, framework synthesis and meta‐ethnography are Cochrane's recommended methods to produce syntheses that can subsequently be integrated with an intervention review (Noyes et al., 2018, [1], Glenton et al., 2021a, [45]). Among the 31 reviews, the majority reported using thematic synthesis (*n* = 14), followed by framework synthesis (*n* = 9), meta‐ethnography (*n* = 3), narrative synthesis (*n* = 2), and realist evaluation (*n* = 1). One review did not specify the methodology. Dennett (2021) only included one qualitative study and was therefore unable to apply synthesis methods (Figure 9). However, it must be noted that some reviews while describing thematic synthesis as their QES methodology referred to using thematic analysis which is the analysis of raw data as opposed to secondary data. Similarly, on occasion reviews referred to using framework analysis which also consists of the analysis of raw data while describing framework synthesis.

Of 11 MMRs, five (45.5%) adequately described the methods for both QES and intervention effectiveness components. Three (27.2%) MMRs could have provided more detail of the methods for both the QES component and intervention effectiveness. One (9.1%) provided an incomplete description of the methods used and two (18.2%) did not report the methods for both the QES and intervention effectiveness components [42, 43]. Many review authors reported using existing frameworks (e.g. SURE framework) to inform data extraction whilst others [16], reported adapting existing guidance such as EPOC or designing their own data extraction form or framework [17, 30]; Dennet et al., 2021; [31]). For instance, Agarwal et al. [16] reported that they adapted the EPOC standard to inform the extraction of study characteristics and outcome data. Similarly, Merner et al. [34] reported that they adapted the Cochrane Consumers and Communication's Data template and the NICE (2012) Examples of Evidence Tables to create a data extraction framework. However, no information was provided regarding the type of qualitative data extracted from their study findings.

**Figure 10:**
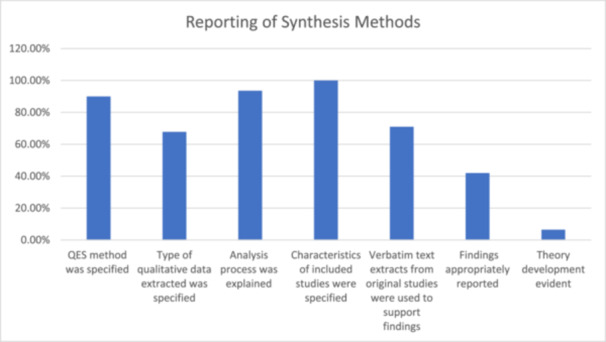
**Percentage of reviews that achieved a green assessment for indicators relating to reporting of synthesis methods**.

Two (18%) of the MMRs were not clear regarding their data analysis processes, one [41] provided little information and the other [35] did not specify the methodology used. Specifically, Vasudevan et al. [41] explained in the synthesis section that the SURE Framework was used to inform the extraction of themes from included studies. The authors used this method in the context of digital interventions for health systems strengthening. However, the authors did not explain how the data were synthesised to generate the new themes in their review. Mischke et al. [35] explained their use of GRADEpro to present qualitative data, however, it is not clear how the authors synthesised the data. Notably, GRADEpro was designed to support the development of guidelines using quantitative evidence. Dennett et al. [22] planned to use thematic synthesis to synthesise the qualitative data in their MMR, however, the authors only included one qualitative study and were unable to perform any type of analysis (see. Figure 10).

### 3.6 Reporting qualitative findings and theory development

All reviews (*n* = 31; 100%) reported characteristics of included studies. Twenty‐one (68%) specified the type of qualitative data extracted, for example themes and concepts including participant quotations, and/or authors' understandings. Two (6%) provided some information [15, 36], three (10%) provided very little information [26, 27, 32] and four (13%) did not specify what type of data from individual studies were extracted [16, 17, 34, 35]. Ames et al. [17] described extracting key themes and categories relevant to synthesis objectives without specifying if participant quotations, and/or authors' understandings were extracted.

The reporting of the synthesis product that aligned with the method of synthesis used lacked clarity. Most review reports defaulted to reporting descriptive level themes without further consideration of what the specific synthesis method was intended to produce (e.g. new frameworks, analytical level themes, lines of argument, new theories and models). As recommended by Flemming et al. [1] most reviews (*n* = 22; 71%) included verbatim text extracts from primary studies to support their findings, however seven (23%) did not include extracts and two (6%) included few extracts [15, 40]. Only thirteen reviews (42%) reported findings using themes, concepts, or interpretation, as well as providing summarised statements of the findings to which GRADE‐CERQual could be applied. Ten reviews (32%) reported some themes and verbatim text extracts but lacked interpretations; but also provided summarised statements of the findings to which GRADE‐CERQual could be applied. Five reviews (16%) [16, 19], Jorden et al., 2016, Munabi‐Babigumira et al., 2017, Thompson et al., 2013) reported themes and summarised statements for GRADE‐CERQual without text extracts or interpretation, while the remaining three (10%; [18, 26, 41]) lacked clear themes and/or presented their findings in an unstructured format, such as only using the types of summarised statements to which GRADE‐CERQual could be applied.

The reporting of theory development was not evident in most reviews; this was absent in 18 reviews (58%) with one (3%) having an incomplete description [40]. Only two reviews (6%) effectively reported theory development [38, 39]. One possible explanation for this difference is the type of methodology used. For instance, one of the two reviews reporting theory development utilised a realist synthesis [38], a theory‐driven approach explicitly designed to develop, test, and refine theoretical frameworks [46]. In contrast, Taylor et al. [39] used thematic synthesis, which is typically descriptive in nature. However, the authors achieved a notable degree of analytical transformation, progressing beyond the initial thematic findings to develop a conceptual model. This suggests that while some methodologies are inherently aligned with theory development, others may also facilitate theoretical insights when applied with an interpretive focus and sufficient depth of analysis. The remaining 10 reviews (32%) included the development of logic models, conceptual models or similar theory development, but would have benefitted from greater detail or clarity (Figure 10). Another distinction is that most reviews used theory to inform their reviews but did not further develop these theories, whereas two reviews used synthesis methods to enable the development of new theory/theoretical insights that went beyond the findings of the included primary studies to develop new interpretations and new findings.

### 3.7 Reporting integration with intervention effect review

**Figure 11:**
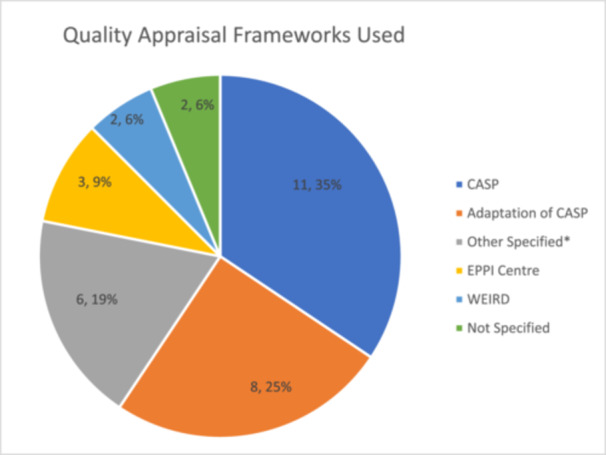
Quality appraisal frameworks used. *Other specified included: Wallace Criteria, Walsh 2006, Thomas 2008, Popay 1998.

Thirteen reviews (42%) integrated their findings with an intervention effect review, 11 (85%) of which clearly explained the method used for integration and two (15%; [27, 30]) could have provided a clearer or more detailed explanation. Of those reviews that reported the integration of their findings with an intervention effect review, the most popular method for integration was a matrix (*n* = 7; 54%), other methods reported were logic models (*n* = 2; 14%; [19, 31]), a conceptual framework (*n* = 1; 7%; [30]), and an integrative review (*n* = 1; 7%; [29]). Odendaal et al. [36] used GRADE evidence‐to‐decision tables to integrate their review with the findings of six Cochrane reviews of effectiveness. Two (15%; [19, 29]) reported using more than one method of integration. For instance, Bohren et al. [19] developed a QES to explain and contextualise the findings from a related review of interventions. The authors reported using a logic model and a matrix to integrate the synthesised qualitative findings with the intervention review. The authors reported using an iterative process to develop logic models depicting theories and hypotheses about the links between findings based on the evidence from both reviews. The authors reported using the ‘Summary of qualitative findings’ from GRADE CERQual to identify features from the findings which were then organised into groups using a matrix‐model approach (Figure 11).

### 3.8 Reporting quality assessment and confidence in the review findings

Most reviews included primary study quality assessments (*n* = 29; 94%), two (6%; [26, 35]) did not report using any assessment. Most reviews (*n* = 11; 35%) used the Critical Appraisal Skills Programme (CASP) tool, or an adaptation of CASP (*n* = 8; 26%; [15, 17, 19, 21], Glenton et al., 2021a, [12, 36, 47]), two (6%) applied the Wallace Criteria [20, 30], two (6%) used the Ways of Evaluating Important and Relevant Data (WEIRD) tool [16, 41], three (9%) used standardised criteria developed by the EPPI‐centre [24, 29, 39], and one applied the criterion from Walsh and Downe [48] D [23]). Thomson et al. [40] adapted the quality appraisal tool developed by Thomas (2008). Jordan et al. [31] used both CASP and adaptations of a framework developed by Popay (1998), with guidance provided by the Cochrane's Qualitative and Implementation Methods Group (QIMG) to develop a quality appraisal form. Jordan et al. [31] adopted a multidimensional concept of quality to assess domains related to the quality of reporting, the methodological rigour, and the conceptual integrity (Figure 11).

Most of the reviews reported confidence in the review findings appropriately (n = 26; 84%) using GRADE CERQual. Hurley et al. [29] and Agarwal et al. [16] used both GRADE (for intervention effects) and GRADE CERQual (for qualitative findings) to assess the confidence of findings from their mixed‐methods syntheses. Three review authors (10%) did not perform any assessment of confidence for their qualitative findings [30, 35, 40] two of which were published in advance of GRADE CERQual being published in 2015.

### 3.9 Reporting reflexivity and limitations

**Figure 12:**
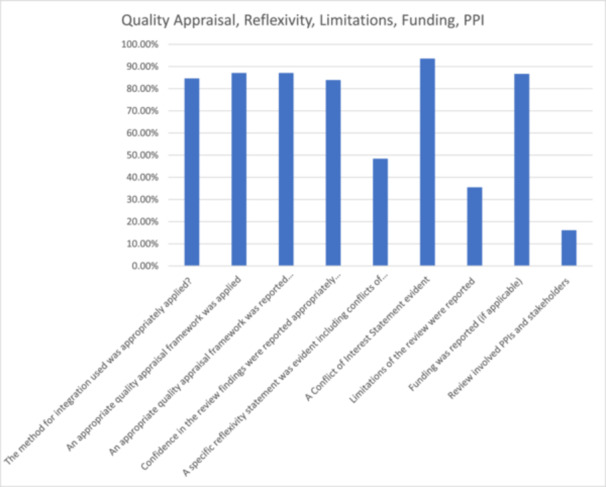
**Percentage of reviews that achieved a green assessment for individual indicators in relation to quality appraisal, reflexivity, limitations, funding, patient and public involvement**.

Less than half of reviews (*n* = 15, 48%) included a comprehensive reflexivity statement, 10 (32%) did not include any statement, four (13%) included some information [17, 18, 20, 23] and two (6%) included very little information [16, 22]. Some mixed‐methods syntheses reported reflexivity in the section describing ‘potential bias in the review process.’ Most reviews included a comprehensive conflict of interest statement (*n* = 29; 94%), one (3%) provided some information in relation to conflict of interest [27] and one (3%) [26] provided little information. Eleven (35%) reviews included full descriptions of their limitations, however nine (29%) did not include limitations, seven (23%) included a moderate amount of information and four (13%) (Glenton et al., 2021a, [26, 35, 39]) included little information (Figure 12).

### 3.10 Reporting funding, stakeholder or patient and public involvement

Review funding was clearly reported in 26 reviews (87%) but was unclear in four (13%; [18, 26, 27, 28]). The reporting of sources of funding varied, with some lacking detail (Figure 12).

Stakeholder involvement or PPI was only reported in five reviews (16%; Glenton et al., 2021a, [28, 30, 34, 38]), one review needed more clarity (3%; [31]) and it was not apparent in the remaining 25 reviews (81%). PPI reporting guidance suggests details should be reported in the aims, methods, results, discussion, and conclusion, and include critical reflections on what worked and what didn't work regarding the involvement activities [49]. Where stakeholder involvement was reported, stakeholders were primarily healthcare professionals or other practitioners. Involvement varied, with some authors [30] involving stakeholders in discussions about contextual factors likely to influence the findings of the review, while others involved PPI members in providing feedback on implications for practice (Glenton et al., 2021a, [28]), or in review processes [30]. Specifically, Merner et al.'s (2023) review was co‐produced with professional stakeholders, who screened papers for inclusion, discussed the findings, and developed principles for best practice, with some qualifying for coauthorship of the paper.

One review, by Jordan et al. [31] involved two consumer referees in the preparation of the protocol and review, but no further detail was provided regarding these individuals and the nature of their expertise, or the precise extent and nature of their involvement (Figure 12).

## 4 DISCUSSION

Overall, our findings highlight gaps in the quality of Cochrane QES reporting, indicating the need for further guidance specifically tailored for both QESs and MMRs with a qualitative component. We produced a composite reporting framework drawing on three commonly used reporting templates. If one of these reporting templates was used by review authors in isolation, it was unlikely to satisfy the essential elements of a well reported QES or MMR with a qualitative component in sufficient detail. Just over a quarter of 31 reviews were considered to provide good quality and detailed descriptions in their reporting based on criteria within our framework, all of which were published since 2019. Most of the remainder required more detail or clarity, particularly regarding reporting of the full synthesis findings that align with the synthesis method used, equity perspective, question formulation, reflexivity statements, funding, and PPI. Many reviews would have benefitted from more complete reporting of findings, particularly the inclusion of interpretation and of verbatim text extracts to support the themes presented. This is similar to France et al.'s (2014) review of meta‐ethnography reporting quality a decade ago, where they reported that the reporting of the synthesis product that aligned with the method of synthesis used in papers in their review also lacked clarity.

Recognising that a new PRISMA version for QES is required, the UK Medical Research Council in 2024 has funded a methodological project to develop PRISMA‐QES and this will assess the degree of fit of PRISMA 2020 across a diverse sample of QES. This will address many of these reporting gaps as the PRISMA guideline is being specifically designed for QES (PRISMA QES, [50]). Once published, this guideline is expected to provide comprehensive and standardised reporting recommendations for QESs, alleviating the current burden on review teams to synthesise multiple templates or create their own reporting frameworks. This will likely enhance clarity, consistency, and quality in QES reporting. However, until this guideline becomes widely adopted, review authors must be encouraged to use the most applicable current templates (e.g., eMERGe, ENTREQ, EPOC) in combination to ensure comprehensive reporting. Likewise, journal editors will be challenged to ensure QES and MMR papers with qualitative components have considered a combination of these reporting templates.

Similar to France et al.'s (2014) review, our review showed that most papers explicitly and fully reported methods for literature searching, databases searched, quality assessment and selecting studies for inclusion. Most reviews appeared to have performed and reported quality assessments of methodological strengths and limitations of primary studies appropriately, albeit in the absence of a specific appraisal tool for QES. Recently, the development of a new evidence‐based tool for assessing the methodological strengths and limitations of primary qualitative studies in a QES ‘CochrAne qualitative MEthodological LimitatiOns Tool’ (CAMELOT) addresses this gap [51]. When GRADE CERQual became available, it was quickly adopted by review authors which is a key tool for facilitating the use of qualitative evidence in decision making processes [52]. A recent evaluation of GRADE‐CERQual fidelity confirmed that this approach is commonly applied well in Cochrane reviews [53].

Our review shows that informative titles have improved immensely over the last decade. For example, when France et al. [54] examined the reporting quality of reviews using meta‐ethnography, reporting guidance was not available and therefore only 32% of reviews included meta‐ethnography in their titles. One likely explanation for some of the reviews performing insufficiently against certain criteria in our review (e.g., titles) is that these reviews also predated the various reporting standards for QES or the reporting standards and guidance documents themselves need further development. In addition, in the absence of a single comprehensive PRISMA type of reporting guideline that could be applied to QESs and MMRs with a qualitative component, review authors did not seem to draw on all available reporting guidance for these review types. It was likely too high a burden for each review team to develop their own synthesis of reporting domains derived from the available commonly used reporting templates, which have been published at different timepoints over the last 12 years. Similarly, guidance for reporting equity in research is only recently available [55].

Another noteworthy finding was that the reporting of stakeholder involvement appeared to consist of professionals only, a description of the characteristics of consumer referees was missing in one review [31]. PPI in QES is essential to ensure that the question and outcomes are relevant and meaningful to those affected by the research [56]. Guidance for improving the reporting of such involvement has been published [49]. These standards emphasise the importance of equality, partnership, and inclusivity in involving the public in research and reporting is important for transparency.

Finally, the lack of specific research questions and/or explicit reported use of a framework to develop a question, in most of the included reviews was surprising. This is likely to be a result of review authors having to initially shoehorn their review reporting into the REVMan template for publishing intervention reviews. This template did not have a requirement for reporting a review question and instead focussed on reporting the PICO and review objectives. More recently, a more flexible REVMan template was produced for reporting QESs and MMRs with a qualitative component, but review authors have not sufficiently changed their reporting practice to align more closely with agreed conventions for reporting these specific types of reviews. Review question formulation is essential to guide and develop methods, including eligibility criteria and search strategies. Use of a framework to structure the research questions frames the entire research process, determining the scope of the review whilst providing a focus [57]. Common question frameworks used for systematic reviews typically employ an epidemiological design (e.g. PICO), which is particularly suited to experimental or observational epidemiological models [58]. These question frameworks are incompatible with QES, and alternatives have been proposed (e.g. PerSPEcTiF; [57]). Although most reviews did not explicitly mention using a particular question framework, their methods and research implicitly indicated adherence to structure. This suggests that the challenge may stem from a lack of suitable reporting guidance regarding question frameworks for QES.

## 5 STRENGTHS AND LIMITATIONS

To the best of our knowledge, this is the first attempt to assess QESs and MMRs with a qualitative component published in the Cochrane Library using a composite framework to assess the quality of reporting. Given that the framework was developed by the research team it warrants future validation, both in terms of specific criteria used, and the overall judgement. While the parameters were not externally validated, expert opinion matched the expert judgement of the reporting quality of the results. The three selected reporting guidelines (the EPOC guidance, ENTREQ and eMERGe) were designed for reporting QES and cited in Cochrane author guidance. We did not include PRISMA 2020 domains in the integrated framework as it was not designed for reporting QES. Noyes, Booth and Harden (QIMG convenors and co‐authors) had previously undertaken an assessment of PRISMA 2020 domains and concluded that most domains needed adaption or were not applicable to a QES context. The few PRISMA 2020 domains that could be applied without adaptation were already covered by the three included reporting guidelines. International expert input and agreement was sought, which strengthens our confidence in the coding approach used. The researchers evaluated review reporting quality individually, with identified discrepancies subsequently deliberated through re‐reviewing the study in collaboration. The framework was also piloted and refined in advance by coding one review in triplicate, to calibrate the coding process. Coding and data analysis were completed in duplicate to mitigate the risk of errors or bias.

## 6 CURRENT COCHRANE DEVELOPMENTS IN THE PRODUCTION PIPELINE

Along with production of the new Cochrane‐Campbell Handbook for Qualitative Evidence Synthesis, a new interim REVMan template and guidance for QES has been produced that assimilates best practice principles from existing reporting guidelines. These include adapting and incorporating PRISMA reporting principles, and expert opinion as appropriate. This will help ensure the full synthesis product is reported as intended and aligned with the method of synthesis used. In future, review authors should only use this REVMan template and composite guidance when publishing Cochrane reviews. In addition, to align with Cochrane's adoption of PRISMA and the associated extensions as the main reporting guideline feeding into the reporting of Cochrane reviews in REVMan, the development of PRISMA QES commenced late 2024 funded by the UK Medical Research Council. Once PRISMA QES is available, the REVMan template for QES will be further updated. Presently, there is not a REVMan template for MMR with a qualitative component, but review authors will be able to draw on the intervention, QES template headings and guidance to report this specific type of review.

## 7 CONCLUSION

The publication of QESs and MMRs with a qualitative component are relatively new Cochrane review types. As such, there has been much less experience of reporting and publishing these reviews in Cochrane to date. There has been extensive methodological development and innovation of methods and tools for conducting these specific review types, however, current reporting guidelines do not consistently reflect these developments. Review authors have not had access to composite reporting guidance that covers all the reporting conventions and domains considered important.

As part of the reorganisation and evolution of publication processes, Cochrane has invested in the production of new resources to better support the publication of all review types, including QESs and MMRs with a qualitative component.

## AUTHOR CONTRIBUTIONS

**Martina Giltenane**: Conceptualization; data curation; formal analysis; investigation; methodology; project administration; validation; visualization; writing—original draft; writing—review and editing. **Aoife O'Mahony**: Conceptualization; data curation; formal analysis; investigation; methodology; project administration; validation; visualization; writing—original draft; writing—review and editing. **Mayara S Bianchim**: Formal analysis; methodology; project administration; validation; writing—original draft; writing—review and editing. **Andrew Booth**: Writing—review and editing. **Angela Harden**: Writing—review and editing. **Catherine Houghton**: Writing—review and editing. **Emma F France**: Writing—review and editing. **Kate Flemming**: Writing—review and editing. **Katy Sutcliffe**: Writing—review and editing. **Ruth Garside**: Writing—review and editing. **Tomas Pantoja**: Writing—review and editing. **Jane Noyes**: Conceptualization; validation; writing—review and editing.

## CONFLICT OF INTEREST STATEMENT

The authors declare no competing financial interests. Noyes is a member of the Cochrane Editorial Board, Methods Executive, Convenor of the Qualitative and Implementation Methods Group (QIMG). Noyes and Harden are Editors of the Cochrane‐Campbell Handbook of Qualitative Evidence Synthesis. Booth, Harden, Houghton, France, Ames, Flemming, Sutcliffe and Garside are QIMG convenors and Bianchim, Giltenane and O'Mahony are QIMG interns.

## PEER REVIEW

The peer review history for this article is available at https://www.webofscience.com/api/gateway/wos/peer-review/10.1002/cesm.70023.

## Data Availability

All data generated or analysed during this study are included in this published article.

## References

[1] Flemming K , Booth A , Garside R , Tunçalp Ö , Noyes J . Qualitative evidence synthesis for complex interventions and guideline development: clarification of the purpose, designs and relevant methods. BMJ Glob Health. 2019;4:e000882. 10.1136/bmjgh-2018-000882 PMC6350756 30775015

[2] Soilemezi D , Linceviciute S . Synthesizing qualitative research: reflections and lessons learnt by two new reviewers. Int J Qual Methods. 2018;17(1):68014.

[3] Noyes J , Booth A , Cargo M , et al. Cochrane qualitative and implementation methods group guidance series ‐ paper 1: introduction. J Clin Epidemiol. 2018a;97:35‐38. 10.1016/j.jclinepi.2017.09.025 29242094

[4] Noyes J , Booth A , Flemming K , et al. Cochrane qualitative and implementation methods group guidance series—paper 3: methods for assessing methodological limitations, data extraction and synthesis, and confidence in synthesized qualitative findings. J Clin Epidemiol. 2018b;97:49‐58. 10.1016/j.jclinepi.2017.06.020 29247700

[5] Moher D , Shamseer L , Clarke M , et al. Preferred reporting items for systematic review and meta‐analysis protocols (PRISMA‐P) 2015 statement. Syst Rev. 2015;4(1):1. 10.1186/2046-4053-4-1 25554246 PMC4320440

[6] Panic N , Leoncini E , de Belvis G , Ricciardi W , Boccia S . Evaluation of the endorsement of the preferred reporting items for systematic reviews and meta‐analysis (PRISMA) statement on the quality of published systematic review and meta‐analyses. PLoS One. 2013;8(12):e83138. 10.1371/journal.pone.0083138 24386151 PMC3873291

[7] Page MJ , Moher D , Bossuyt PM , et al. PRISMA 2020 explanation and elaboration: updated guidance and exemplars for reporting systematic reviews. BMJ. 2021;372:n160. 33781993 10.1136/bmj.n160 PMC8005925

[8] Tong A , Flemming K , McInnes E , Oliver S , Craig J . Enhancing transparency in reporting the synthesis of qualitative research: ENTREQ. BMC Med Res Methodol. 2012;12:181. 10.1186/1471-2288-12-181 23185978 PMC3552766

[9] France EF , Cunningham M , Ring N , et al. Improving reporting of meta‐ethnography: the eMERGe reporting guidance. BMC Med Res Methodol. 2019;19:25. 10.1186/s12874-018-0600-0 30709371 PMC6359764

[10] Glenton C , Lewin S , Downe S , et al. Qualitative evidence syntheses within cochrane effective practice and organisation of care: developing a template and guidance. Int J Qual Methods. 2021b;20:41959.

[11] Flemming K , Noyes J . Qualitative evidence synthesis: where are we at? Int J Qual Methods. 2021;20:3276.

[12] Glenton C , Colvin CJ , Carlsen B , et al. Barriers and facilitators to the implementation of lay health worker programmes to improve access to maternal and child health: a qualitative evidence synthesis. Cochrane Database Syst Rev. 2013;2013(10):CD010414. 10.1002/14651858.CD010414.pub2 24101553 PMC6396344

[13] PICO Portal . PICO Portal 2020. https://picoportal.org/

[14] Cragun D , Pal T , Vadaparampil ST , Baldwin J , Hampel H , DeBate RD . Qualitative comparative analysis: a hybrid method for identifying factors associated with program effectiveness. J Mixed Methods Res. 2016;10(3):251‐272. 10.1177/1558689815572023 PMC4941817 27429602

[15] Ames HMR , Glenton C , Lewin S , Tamrat T , Akama E , Leon N . Clients' perceptions and experiences of targeted digital communication accessible via mobile devices for reproductive, maternal, newborn, child, and adolescent health: a qualitative evidence synthesis. Cochrane Database Syst Rev. 2019;2019(10):CD013447. 10.1002/14651858.CD013447 PMC6791116 31608981

[16] Agarwal S , Glenton C , Henschke N , et al. Tracking health commodity inventory and notifying stock levels via mobile devices: a mixed methods systematic review. Cochrane Database Syst Rev. 2020;2020(10):CD012907. 10.1002/14651858.CD012907.pub2 PMC8094928 33539585

[17] Ames HMR , Glenton C , Lewin S . Parents' and informal caregivers' views and experiences of communication about routine childhood vaccination: a synthesis of qualitative evidence. Cochrane Database Syst Rev. 2017;2017(2):CD011787. 10.1002/14651858.CD011787.pub2 PMC5461870 28169420

[18] Anglemyer A , Moore THM , Parker L , et al. Digital contact tracing technologies in epidemics: a rapid review. Cochrane Database Syst Rev. 2020;2020(8):CD013699. 10.1002/14651858.CD013699 PMC8241885 33502000

[19] Bohren MA , Berger BO , Munthe‐Kaas H , Tunçalp Ö . Perceptions and experiences of labour companionship: a qualitative evidence synthesis. Cochrane Database Syst Rev. 2019;2019(3):CD012449. 10.1002/14651858.CD012449.pub2 PMC6422112 30883666

[20] Campbell K , Coleman‐Haynes T , Bowker K , Cooper SE , Connelly S , Coleman T . Factors influencing the uptake and use of nicotine replacement therapy and e‐cigarettes in pregnant women who smoke: a qualitative evidence synthesis. Cochrane Database Syst Rev. 2020;2020(5):CD013629. 10.1002/14651858.CD013629 PMC7387757 32441810

[21] Cooper S , Schmidt BM , Sambala EZ , et al. Factors that influence parents' and informal caregivers' views and practices regarding routine childhood vaccination: a qualitative evidence synthesis. Cochrane Database Syst Rev. 2021;2021(10):CD013265. 10.1002/14651858.CD013265.pub2 PMC8550333 34706066

[22] Dennett EJ , Janjua S , Stovold E , Harrison SL , McDonnell MJ , Holland AE . Tailored or adapted interventions for adults with chronic obstructive pulmonary disease and at least one other long‐term condition: a mixed methods review. Cochrane Database Syst Rev. 2021;7(7):CD013384. 34309831 10.1002/14651858.CD013384.pub2 PMC8407330

[23] Downe S , Finlayson K , Tunçalp Ö , Gülmezoglu AM . Provision and uptake of routine antenatal services: a qualitative evidence synthesis. Cochrane Database Syst Rev. 2019;2019(6):CD012392. 10.1002/14651858.CD012392.pub2 PMC6564082 31194903

[24] Engel N , Ochodo EA , Karanja PW , et al. Rapid molecular tests for tuberculosis and tuberculosis drug resistance: a qualitative evidence synthesis of recipient and provider views. Cochrane Database Syst Rev. 2022;2022(4):CD014877. 10.1002/14651858.CD014877.pub2 PMC9038447 35470432

[25] Glenton C , Carlsen B , Lewin S , Wennekes MD , Winje BA , Eilers R . Healthcare workers' perceptions and experiences of communicating with people over 50 years of age about vaccination: a qualitative evidence synthesis. Cochrane Database Syst Rev. 2021;2021(7):CD013706. 10.1002/14651858.CD013706.pub2 PMC8407331 34282603

[26] Horey D , Kealy M , Davey MA , Small R , Crowther CA . Interventions for supporting pregnant women's decision‐making about mode of birth after a caesarean. Cochrane Database Syst Rev. 2013;2013(7):CD010041. 10.1002/14651858.CD010041.pub2 23897547 PMC11608817

[27] Houghton C , Dowling M , Meskell P , et al. Factors that impact on recruitment to randomised trials in health care: a qualitative evidence synthesis. Cochrane Database Syst Rev. 2020a;2020(10):MR000045. 10.1002/14651858.MR000045.pub2 PMC8078544 33026107

[28] Houghton C , Meskell P , Delaney H , et al. Barriers and facilitators to healthcare workers' adherence with infection prevention and control (IPC) guidelines for respiratory infectious diseases: a rapid qualitative evidence synthesis. Cochrane Database Syst Rev. 2020b;2020(4):CD013582. 10.1002/14651858.CD013582 PMC7173761 32315451

[29] Hurley M , Dickson K , Hallett R , et al. Exercise interventions and patient beliefs for people with hip, knee or hip and knee osteoarthritis: a mixed methods review. Cochrane Database Syst Rev. 2018;2018(4):CD010842. 10.1002/14651858.CD010842.pub2 PMC6494515 29664187

[30] Husk K , Lovell R , Cooper C , Stahl‐Timmins W , Garside R . Participation in environmental enhancement and conservation activities for health and well‐being in adults: a review of quantitative and qualitative evidence. Cochrane Database Syst Rev. 2016;2016(5):CD010351. 10.1002/14651858.CD010351.pub2 27207731 PMC6464867

[31] Jordan J , Rose L , Dainty KN , Noyes J , Blackwood B . Factors that impact on the use of mechanical ventilation weaning protocols in critically ill adults and children: a qualitative evidence‐synthesis. Cochrane Database Syst Rev. 2016;2016(1):CD011812. 10.1002/14651858.CD011812.pub2 PMC6458040 27699783

[32] Karimi‐Shahanjarini A , Shakibazadeh E , Rashidian A , et al. Barriers and facilitators to the implementation of doctor‐nurse substitution strategies in primary care: a qualitative evidence synthesis. Cochrane Database Syst Rev. 2019;2019(4):CD010412. 10.1002/14651858.CD010412.pub2 PMC6462850 30982950

[33] Lins S , Hayder‐Beichel D , Rücker G , et al. Efficacy and experiences of telephone counselling for informal carers of people with dementia. Cochrane Database Syst Rev. 2014;2014(9):CD009126. 25177838 10.1002/14651858.CD009126.pub2 PMC7433299

[34] Merner B , Schonfeld L , Virgona A , et al. Consumers' and health providers' views and perceptions of partnering to improve health services design, delivery and evaluation: a co‐produced qualitative evidence synthesis. Cochrane Database Syst Rev. 2023;2023(3):CD013274. 10.1002/14651858.CD013274.pub2 PMC10065807 36917094

[35] Mischke C , Verbeek JH , Job J , et al. Occupational safety and health enforcement tools for preventing occupational diseases and injuries. Cochrane Database Syst Rev. 2013;2013(8):CD010183. 10.1002/14651858.CD010183.pub2 23996220 PMC11707410

[36] Odendaal WA , Anstey Watkins J , Leon N , et al. Health workers' perceptions and experiences of using mHealth technologies to deliver primary healthcare services: a qualitative evidence synthesis. Cochrane Database Syst Rev. 2020;2020(3):CD011942. 10.1002/14651858.CD011942.pub2 PMC7098082 32216074

[37] Pollock A , Campbell P , Cheyne J , et al. Interventions to support the resilience and mental health of frontline health and social care professionals during and after a disease outbreak, epidemic or pandemic: a mixed methods systematic review. Cochrane Database Syst Rev. 2020;2020(11):CD013779. 10.1002/14651858.CD013779 PMC8226433 33150970

[38] Rivas C , Vigurs C , Cameron J , Yeo L . A realist review of which advocacy interventions work for which abused women under what circumstances. Cochrane Database Syst Rev. 2019;2019(6):CD013135. 10.1002/14651858.CD013135.pub2 PMC6598804 31254283

[39] Taylor M , Thomas R , Oliver S , Garner P . Community views on mass drug administration for filariasis: a qualitative evidence synthesis. Cochrane Database Syst Rev. 2022;2022(2):CD013638. 10.1002/14651858.CD013638.pub2 PMC8851040 35174482

[40] Thomson H , Thomas S , Sellstrom E , Petticrew M . Housing improvements for health and associated socio‐economic outcomes. Cochrane Database Syst Rev. 2013;2013(2):CD008657. 10.1002/14651858.CD008657.pub2 PMC12551615 23450585

[41] Vasudevan L , Glenton C , Henschke N , et al. Birth and death notification via mobile devices: a mixed methods systematic review. Cochrane Database Syst Rev. 2021;2021(7):CD012909. 10.1002/14651858.CD012909.pub2 PMC8785898 34271590

[42] Velez M , Lugo‐Agudelo LH , Patiño Lugo DF , et al. Factors that influence the provision of home‐based rehabilitation services for people needing rehabilitation: a qualitative evidence synthesis. Cochrane Database Syst Rev. 2023;20232:CD014823. 10.1002/14651858.CD014823 PMC9918343 36780267

[43] Yoshino CA , Sidney‐Annerstedt K , Wingfield T , et al. Experiences of conditional and unconditional cash transfers intended for improving health outcomes and health service use: a qualitative evidence synthesis. Cochrane Database Syst Rev. 2023;2023(3):CD013635. 10.1002/14651858.CD013635.pub2 PMC10064639 36999604

[44] Ames HMR , France EF , Cooper S , et al. Assessing qualitative data richness and thickness: development of an evidence‐based tool for use in qualitative evidence synthesis. Cochrane Evidence Synth Methods. 2024;2(7):e12059. 10.1002/cesm.12059 PMC11795969 40475321

[45] Glenton C , Bohren MA , Downe S , Paulsen EJ , Lewin S . on behalf of Effective Practice and Organisation of Care (EPOC). EPOC Qualitative Evidence Synthesis: Protocol and review template. Version 1.3. EPOC Resources for review authors. Norwegian Institute of Public Health; 2022. Available at: http://epoc.cochrane.org/epoc-specific-resources-review-authors

[46] Rycroft‐Malone J , McCormack B , Hutchinson AM , et al. Realist synthesis: illustrating the method for implementation research. Implement Sci. 2012;7:33. 10.1186/1748-5908-7-33 22515663 PMC3514310

[47] Munabi‐Babigumira S , Glenton C , Lewin S , Fretheim A , Nabudere H . Factors that influence the provision of intrapartum and postnatal care by skilled birth attendants in low‐ and middle‐income countries: a qualitative evidence synthesis. Cochrane Database Syst Rev. 2017;2017(11):CD011558. 10.1002/14651858.CD011558.pub2 PMC5721625 29148566

[48] Walsh D , Downe S . Appraising the quality of qualitative research. Midwifery. 2006;22(2):108‐119. 10.1016/j.midw.2005.05.004 16243416

[49] Staniszewska S , Brett J , Simera I , et al. GRIPP2 reporting checklists: tools to improve reporting of patient and public involvement in research. BMJ. 2017;358:j3453. 28768629 10.1136/bmj.j3453 PMC5539518

[50] Preferred Reporting Items for Systematic reviews and Meta‐Analysis (PRISMA) for Qualitative Evidence Synthesis (QES) website (2025[Online] Available from): https://sites.google.com/view/prismaqes/home [Accessed January 2025]

[51] Munthe‐Kaas HM , Booth A , Sommer I , et al. Developing CAMELOT for assessing methodological limitations of qualitative research for inclusion in qualitative evidence syntheses. Cochrane Evidence Synth Methods. 2024;2(6):e12058. 10.1002/cesm.12058 PMC11795985 40475879

[52] Lewin S , Booth A , Glenton C , et al. Applying GRADE‐CERQual to qualitative evidence synthesis findings: introduction to the series. Implement Sci. 2018;13(suppl 1):2. 10.1186/s13012-017-0688-3 29384079 PMC5791040

[53] Wainwright M , Zahroh RI , Tunçalp Ö , et al. The use of GRADE‐CERQual in qualitative evidence synthesis: an evaluation of fidelity and reporting. Health Res Policy Syst. 2023;21(1):77. 10.1186/s12961-023-00999-3 37491226 PMC10369711

[54] France EF , Ring N , Thomas R , Noyes J , Maxwell M , Jepson R . A methodological systematic review of what's wrong with meta‐ethnography reporting. BMC Med Res Methodol. 2014;14:119. 10.1186/1471-2288-14-119 25407140 PMC4277825

[55] Popay J , Chekar CK , Griffiths A , et al. Strengthening the equity focus of applied public health research: introducing the FOR EQUITY platform. Public Health. 2023;215:12‐16. 10.1016/j.puhe.2022.11.018 36608600

[56] Pollock A , Campbell P , Synnot A , Smith M , Morley R . Patient and public involvement in systematic reviews. in. GIN Public Toolkit: Patient and Public Involvement in Guidelines. Guidelines International Network; 2021.

[57] Booth A , Noyes J , Flemming K , Moore G , Tunçalp Ö , Shakibazadeh E . Formulating questions to explore complex interventions within qualitative evidence synthesis. BMJ Glob Health. 2019;4(suppl 1):e001107. 10.1136/bmjgh-2018-001107 PMC6350737 30775019

[58] Methley AM , Campbell S , Chew‐Graham C , McNally R , Cheraghi‐Sohi S . PICO, PICOS and SPIDER: a comparison study of specificity and sensitivity in three search tools for qualitative systematic reviews. BMC Health Serv Res. 2014;14:579. 25413154 10.1186/s12913-014-0579-0 PMC4310146

